# Aneurysmal Bone Cyst (ABC) of the C2 Vertebra

**DOI:** 10.7759/cureus.27735

**Published:** 2022-08-06

**Authors:** Sook-Kwan Chan, Mohd H Muhamad Ariffin

**Affiliations:** 1 Orthopedics, Hospital Queen Elizabeth, Kota Kinabalu, MYS; 2 Spine Surgery, University Kebangsaan Malaysia Medical Center, Kuala Lumpur, MYS

**Keywords:** cervical spine, c2 vertebra, denosumab, transoral spinal surgery, aneurysmal bone cyst

## Abstract

An aneurysmal bone cyst (ABC) is a benign bone lesion commonly found in the younger age group. The principal aim of complete surgical excision to prevent recurrence in an ABC of the C2 vertebra presents a surgical challenge. We report a case of a 13-year-old with an ABC of the C2 vertebra and the strategies in its management surgically and pharmacologically.

## Introduction

The overall incidence of an aneurysmal bone cyst (ABC) is 1.4% of all primary bone tumors [1]. Its incidence in the vertebral column has been reported from 3-30% of all ABC [1, 2]. Of these vertebral lesions, 22-42% are located at the cervical spine [1]. Challenges that occur when dealing with an ABC located at the cervical spine include the proximity of the spinal cord and the resulting neurological compromise, surgical access in order to achieve adequate excision, and stability of the spine after excision. In this case report, we describe the management of a C2 vertebral ABC in a growing adolescent and discuss the latest treatment options available.

## Case presentation

**Figure 1:**
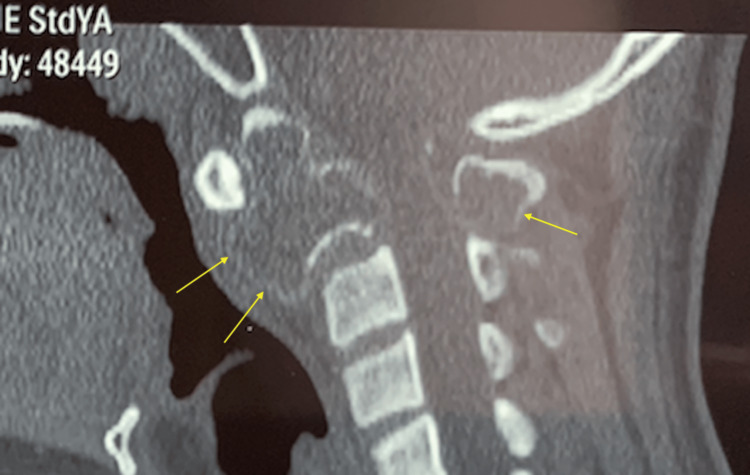
Sagittal CT of the upper cervical spine Yellow arrows show the cortical border of the C2 vertebra which has expanded and thinned out. The lesion has affected the anterior and posterior columns of the vertebra involved.

**Figure 2:**
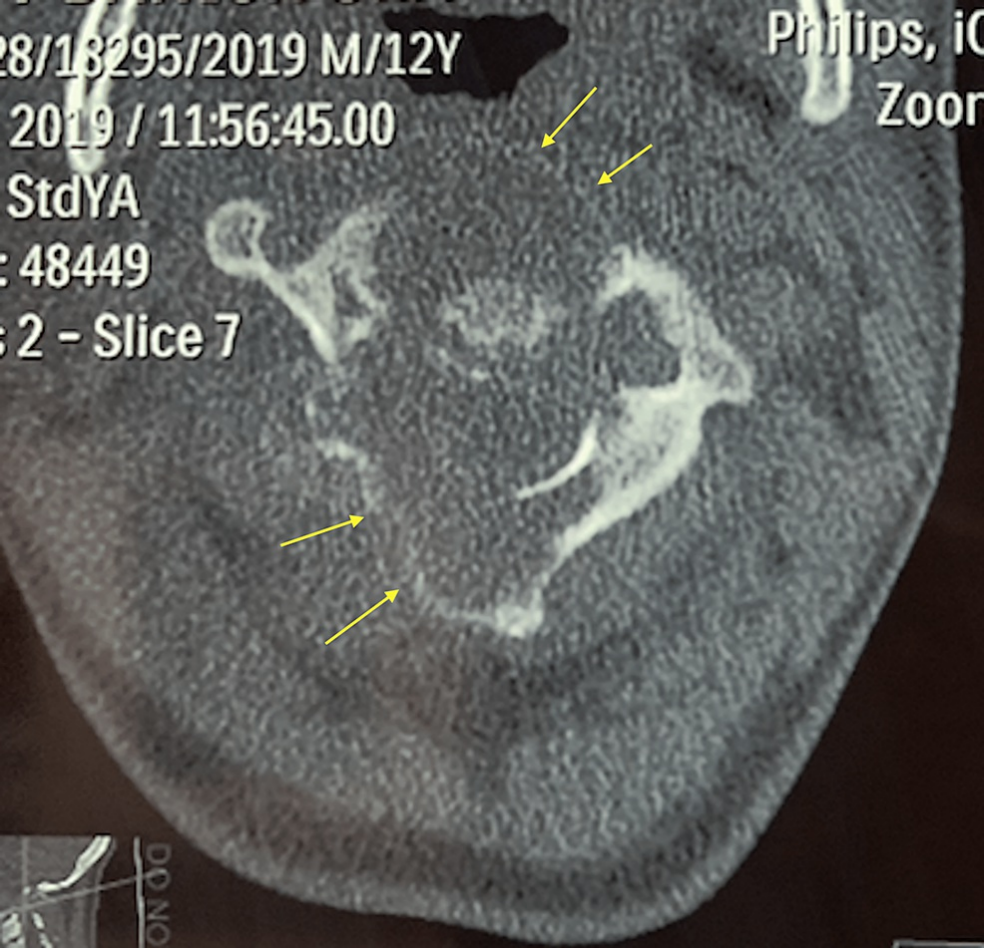
Axial CT of C2 vertebra Yellow arrows show the cortical borders which have been thinned out.

This is a case report of a 13-year-old male with an aneurysmal bone cyst of the C2 vertebra. This patient first presented with persistent and unrelenting neck pain. At his initial clinical assessment on presentation, there were no neurological deficits. MRI of the spine done one month after the onset of symptoms showed an expansile lesion contained within the C2 vertebra involving the anterior and posterior elements (Figure 1, 2). The histological examination of the posterior bony elements of C2 via open biopsy suggested a possibility of a benign bone lesion as no malignant cells were seen. A provisional diagnosis of C2 aneurysmal bone cyst with a differential of giant cell tumor was made based on the imaging and histological finding.

**Figure 3:**
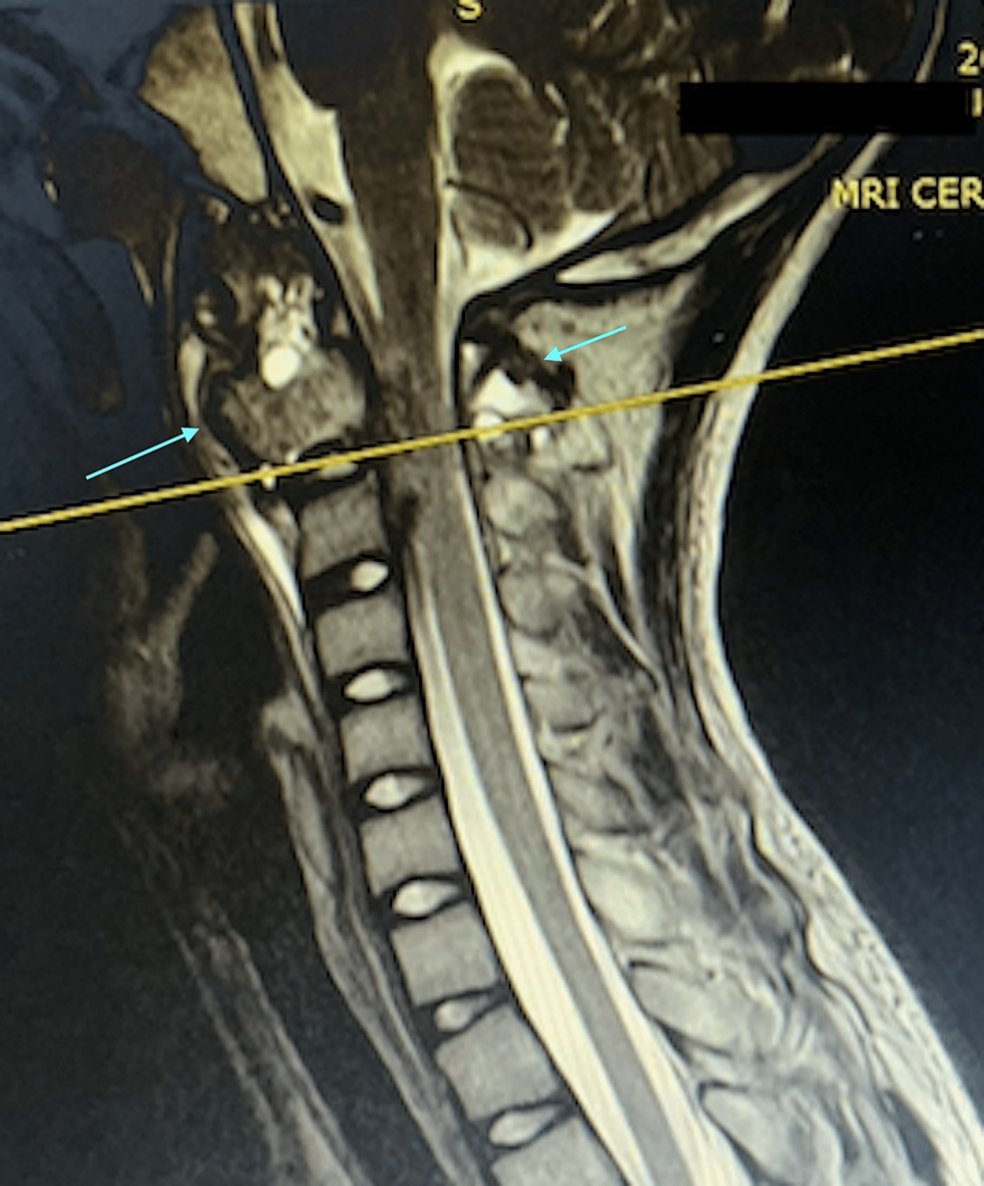
Sagittal MRI of the cervical spine This is the MRI from the time of clinical presentation. The light blue arrows show the lesion contained within the C2 vertebra and involving anterior and posterior columns.

**Figure 4:**
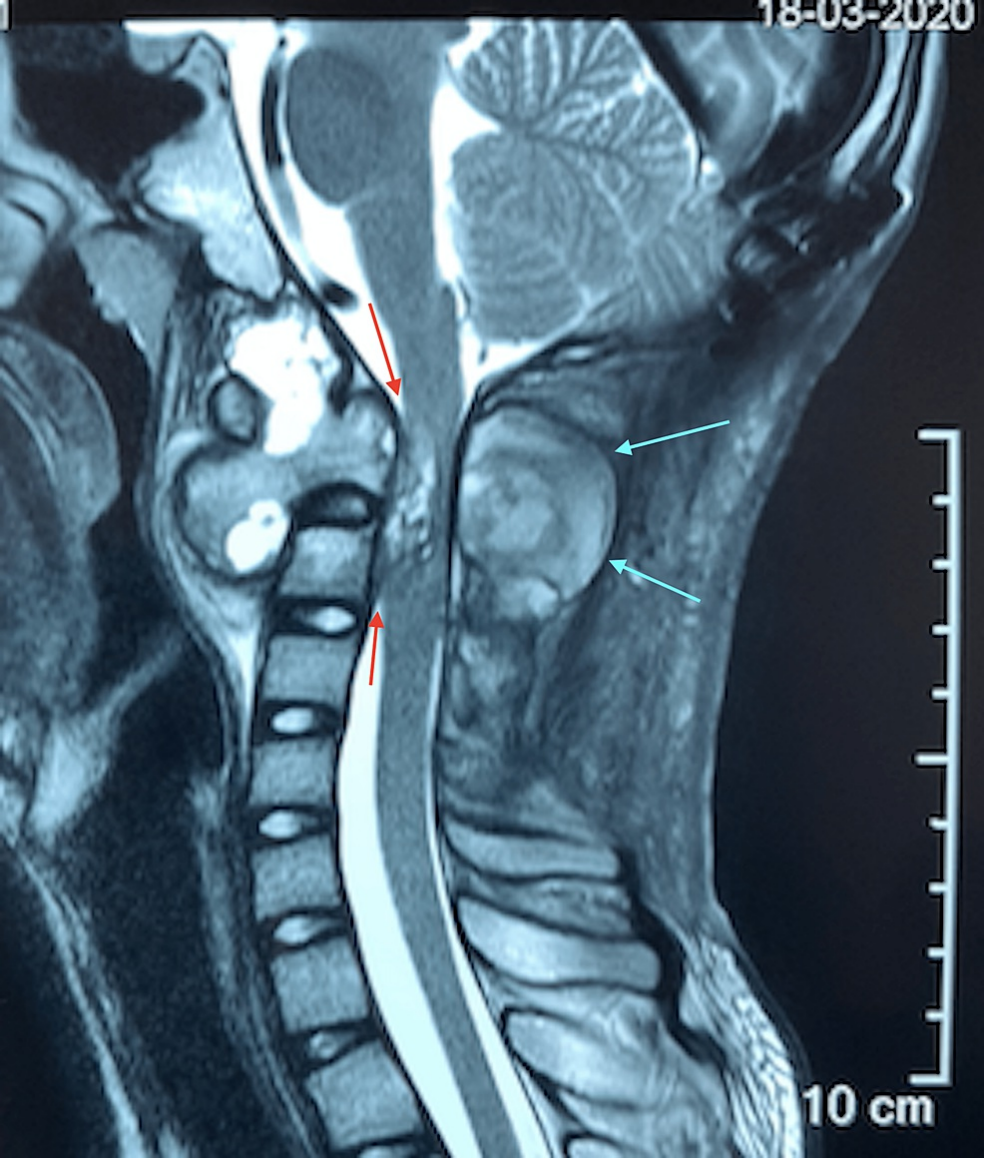
Sagittal MRI of the cervical spine after surgical excision of C2 posterior elements Compared to Figure 3, there is clearly a continued expansion of the lesion anteriorly as well as posteriorly (light blue arrows) despite the previous excision of the posterior elements of C2. This expansion of the lesion has caused more severe narrowing of the spinal canal at this level (red arrows).

Surgical stabilization with posterior instrumentation from occiput to C5 and C2 laminectomy was done from a posterior-only approach. The intra-op bone tissue from this surgery confirmed our provisional diagnosis of an aneurysmal bone cyst. During the planning for the second stage of C2 vertebral body excision and stabilization via anterior approach, the patient developed myelopathy symptoms. MRI post posterior stabilization and laminectomy showed the anterior C2 lesion expanding and compressing on the spinal cord (Figure 3, 4). Oral dexamethasone was started, which resulted in improvement of his myelopathy symptoms while awaiting the second stage via anterior approach to the C2 vertebral body.

**Figure 5:**
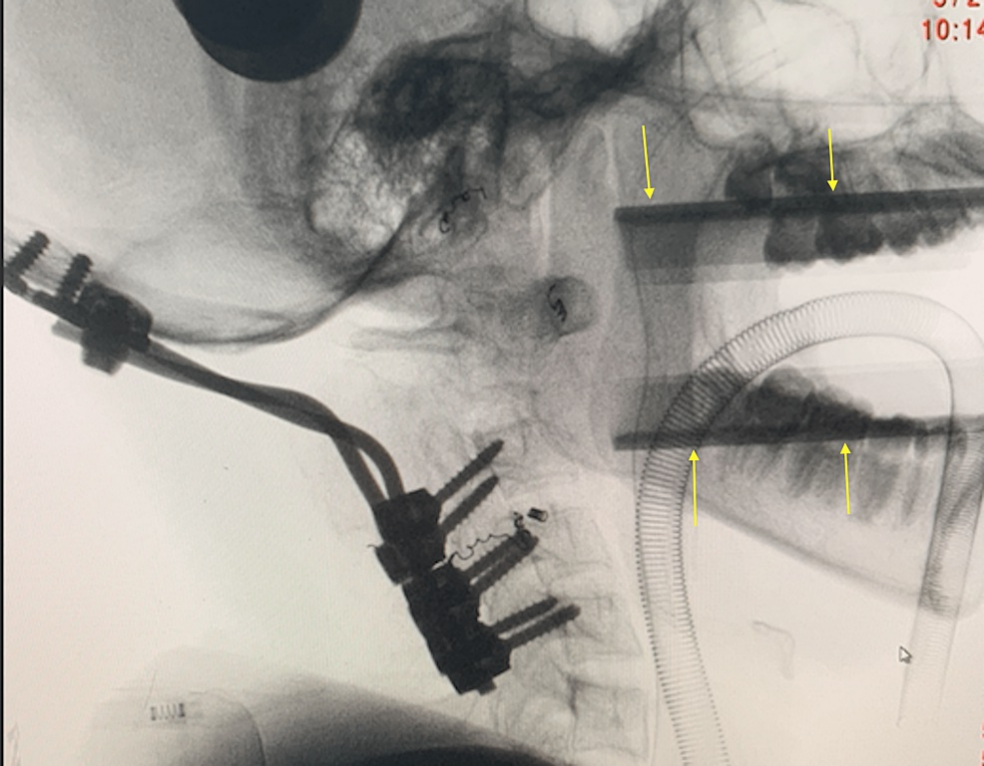
Intra-operative radiography showing access to C2 vertebral body Intra-oral retractors (yellow arrows) are used to visualize the retro-pharyngeal wall for access to the C2 vertebral body.

**Figure 6:**
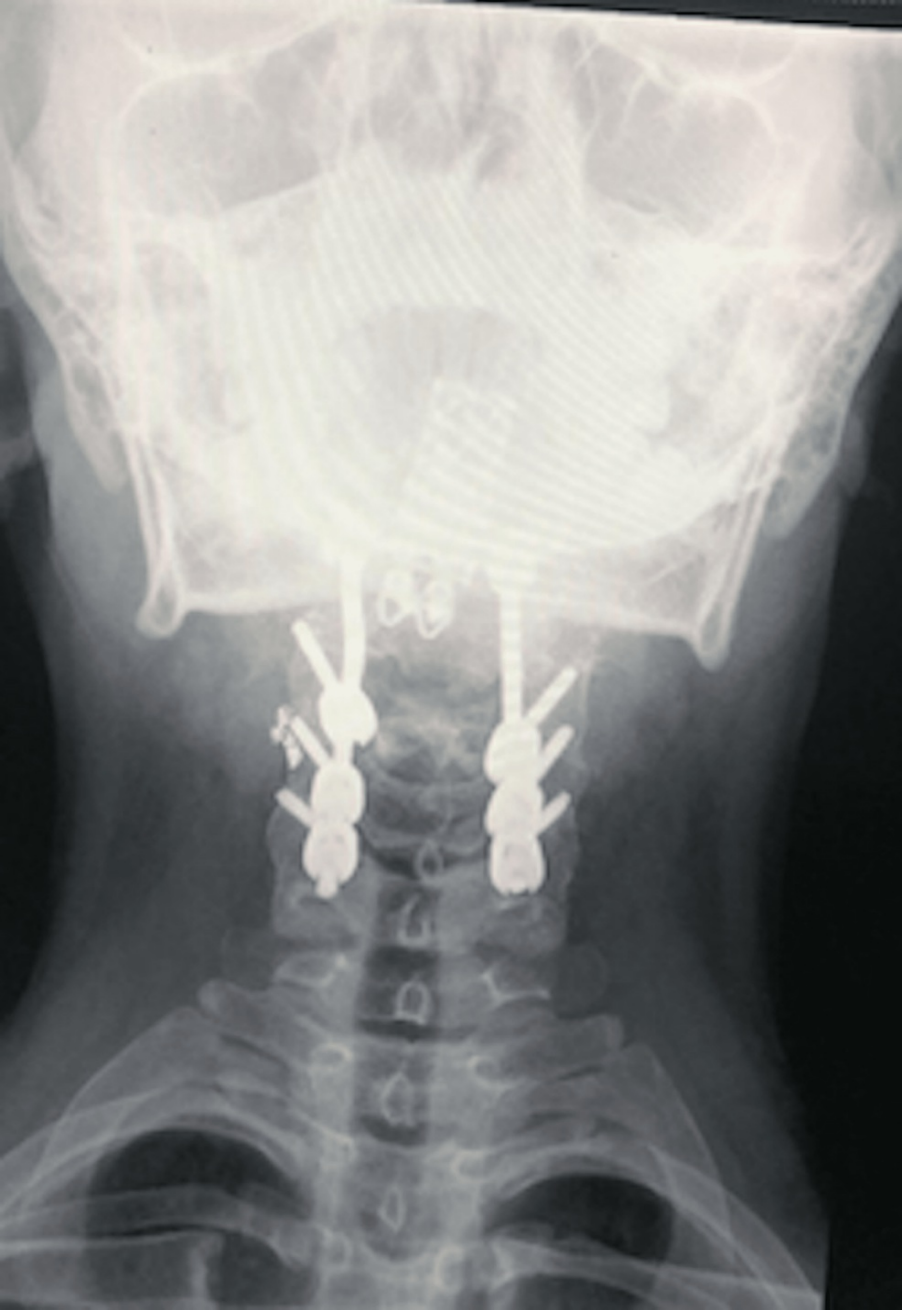
Post-operative anteroposterior view X-ray The posterior fixation is from occiput to C5 using lateral mass screws.

**Figure 7:**
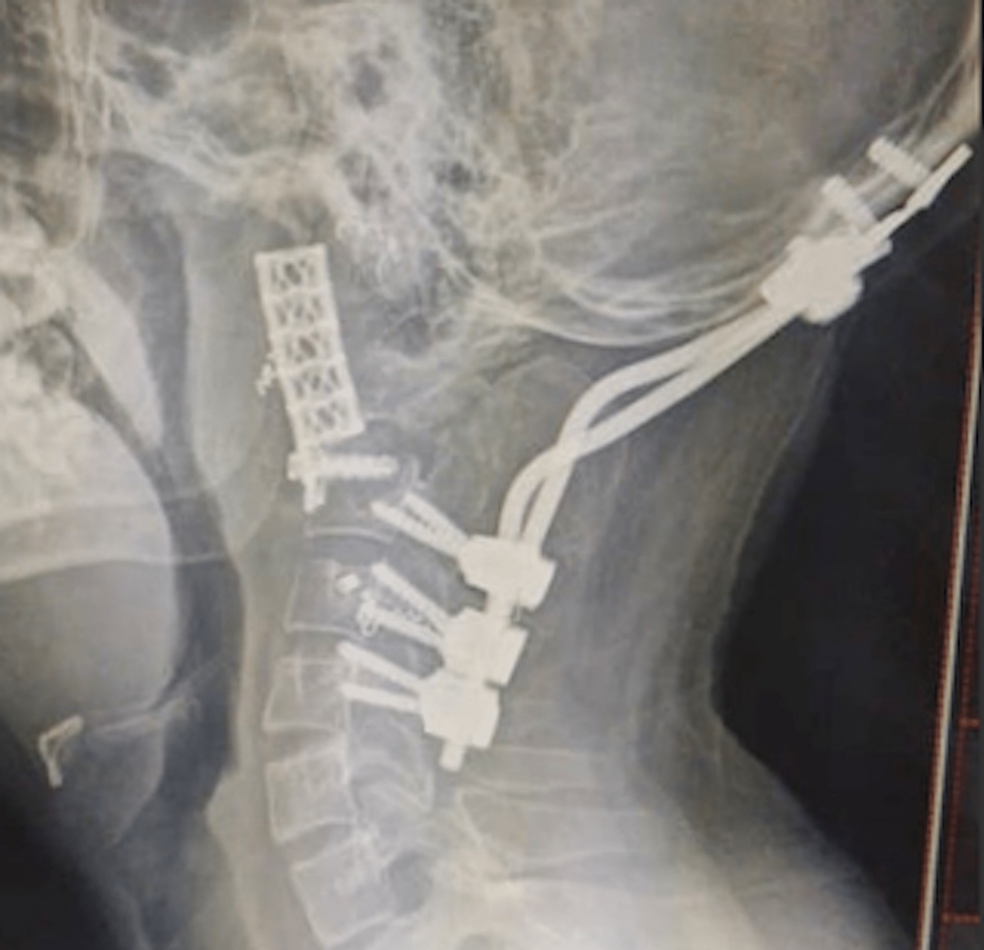
Post-operative lateral view X-ray After anterior excision of C2, the anterior column was stabilized with a mesh cage, with a screw inserted through the cage into the C3 vertebra body.

For the second stage surgery (four months after the posterior stabilization), after a pre-op embolization of the identified feeder arteries to the C2 vertebral body, the patient underwent a trans-oral approach which utilized several intra-oral retractors to aid in the visualization of the retro-pharyngeal wall, where direct access to the C2 body was possible (Figure 5). With this approach, excision of the C2 vertebral body, thus decompression of the spinal canal and anterior stabilization with mesh cage was done (Figure 6, 7). The histological examination from this surgery re-confirmed the tissue diagnosis of an aneurysmal bone cyst. He subsequently received regular monthly denosumab for a total duration of six months (started after the second stage surgery), and at 18 months post surgery for C2 ABC, the patient remains symptom-free, and a repeated CT and MRI showed resolution of the C2 lesion.

## Discussion

From early histological studies of aneurysmal bone cysts, it has been shown to be neoplastic in nature due to evidence of infiltration by the cyst lining. Typical histology of ABC shows endothelium-lined, blood-filled cysts with interstitial fibrous tissue composed of spindle cells and occasional giant cells [3]. While ABC lesions in long bones result in growth disturbance of the limbs due to infiltration of the epiphyseal plate, ABC lesions of the spine, which can infiltrate into the adjacent vertebra or ribs, will result in much graver problems due to the proximity of the spinal cord and neural structures [4]. More than half of the patients with vertebral ABCs present with spinal cord or root compression symptoms [4]. The majority of ABC lesions of the spine will involve the posterior elements (pedicle and lamina), and about one-third will have no involvement of the anterior element (vertebra body) [4]. In our patient, the ABC lesion had involved the entire C2 vertebra even at the time of presentation, as seen on the CT scan(Figure 1, 2). We also saw that despite posterior decompression and laminectomy of C2, due to our inability to excise the anterior part of C2 during the first surgery, the tumor continued to expand, thus resulting in neurological compromise (Figure 3, 4). The natural history of ABC is: firstly, growth by active invasion along blood vessels and passive distension of these blood spaces, then followed by the eventual thrombosis and fibrosis of these vessels and finally the absorption and re-calcification of the lesion [3]. Unfortunately for this patient, due to the progressive compression of the ABC on his spinal cord, we could not wait for natural absorption and re-calcification; therefore, we proceeded with the second stage of the surgery to excise the C2 vertebral body with stabilization via a trans-oral route (Figure 5, 6).

Irradiation therapy alone (without surgical excision) of the ABC lesion has shown to be less effective in tumor control compared to resection (complete or partial) and curettage, which has shown superior outcomes [4]. This less favorable outcome and its association with post-radiation sarcoma transformation has led to the irradiation of ABC becoming a less favorable treatment option. Despite better tumor control with surgical excision and curettage, recurrence rate has been reported at 10-44%, and recurrence will often occur within one to two years post-op [1,5]. Hence the minimum follow-up period post-treatment should be 24 months. Apart from irradiation, another option for ABC lesions with difficult surgical access, such as those located in the cervical spine, is selective arterial embolization (SAE), essentially cutting off the primary blood supply to the lesion and allowing the natural process of absorption and re-calcification to occur. Ideal prerequisites for selective arterial embolization in ABC lesions are: the diagnosis of ABC is certain, no pathological fracture (lesion is intact), and the procedure is technically feasible. This treatment option gives a good cost-to-benefit ratio since if the embolization fails there is a surgical option still available [6]. Our patient, in this case report, underwent a pre-operative embolization (with coils) of the feeding arteries to reduce the intra-operative bleeding and thus facilitate the excision of the lesion; this form of embolization may become the sole treatment option in future spine ABC lesions. An important anatomical factor that needs to be considered when performing SAE is the presence of the anterior spinal artery arising from the same pedicle feeding the lesion; this is a contraindication to its embolization, as this could result in injury to the pyramidal tracts and lead to severe motor deficits [6].

Another non-surgical option is the administration of the drug denosumab (an anti-resorptive acting on the osteoclasts). Molecularly, ABC comprises of osteoclasts-like multinucleated giant cells that express high levels of receptor activator of nuclear kappa B (RANK) receptors and neoplastic stromal cells that express high levels of RANK ligand (RANKL). RANK-RANKL interaction activates a cascade that results in abnormally increased bone resorption. Denosumab is a monoclonal antibody targeted against the RANKL and has been FDA approved to treat giant cell tumors (GCT) of the bone in adults and skeletally mature adolescents. Histologically GCT and ABC share similar features; thus using denosumab in ABC is still being studied for its role in controlling tumor growth [7]. Denosumab use in ABC lesions has been shown to improve pain symptoms and improve the function of the patient over three months of treatment, and 18-82% of the patients showed a reduction in the volume of the lesion on radiological assessment [8]. The reported adverse event of hypercalcemia occurred in two of the nine ABC patients receiving denosumab [8]. Use of denosumab in non-surgically excised, or curetted, ABCs show an 18.6% recurrence rate, a majority of whom were adults [9]. Various regimes and practices have been described in regard to the dosage for denosumab in ABC treatment; one common regime is subcutaneous denosumab 120mg on day one, eight, 15, 29, and then every four weeks with supplementary daily calcium 500mg and vitamin D 400IU [10].

## Conclusions

Although it is uncommon to encounter ABC lesions in the cervical spine, it is important to understand its natural history and the current approaches to treatment. Traditional principles of complete excision may still hold true, but in exceptional cases where the tumours are located in difficult areas to achieve such excision via a safe surgical approach, it is important to be aware of the available options of selective arterial embolization and bisphosphonate (denosumab) treatment to aid in the control of the lesion's growth. Evidently, we were unable to achieve complete excision surgically; thus, the use of post-op denosumab to control the tumor growth until the eventual natural process of absorption and re-calcification of the ABC lesion. This most likely limited the disease progression for this patient and thus we saw no evidence of recurrence at 18 months post-op.
